# Calling Biomarkers in Milk Using a Protein Microarray on Your Smartphone

**DOI:** 10.1371/journal.pone.0134360

**Published:** 2015-08-26

**Authors:** Susann K. J. Ludwig, Christian Tokarski, Stefan N. Lang, Leendert A. van Ginkel, Hongying Zhu, Aydogan Ozcan, Michel W. F. Nielen

**Affiliations:** 1 RIKILT Wageningen UR, Wageningen, The Netherlands; 2 Biologisch-Pharmazeutische Fakultät, Friedrich-Schiller-Universität Jena, Jena, Germany; 3 Electrical Engineering Department, University of California Los Angeles, Los Angeles, California, United States of America; 4 Bioengineering Department, University of California Los Angeles, Los Angeles, California, United States America; 5 California NanoSystems Institute (CNSI), University of California Los Angeles, Los Angeles, California, United States of America; 6 Laboratory of Organic Chemistry, Wageningen University, Wageningen, The Netherlands; Food and Drug Administration, UNITED STATES

## Abstract

Here we present the concept of a protein microarray-based fluorescence immunoassay for multiple biomarker detection in milk extracts by an ordinary smartphone. A multiplex immunoassay was designed on a microarray chip, having built-in positive and negative quality controls. After the immunoassay procedure, the 48 microspots were labelled with Quantum Dots (QD) depending on the protein biomarker levels in the sample. QD-fluorescence was subsequently detected by the smartphone camera under UV light excitation from LEDs embedded in a simple 3D-printed opto-mechanical smartphone attachment. The somewhat aberrant images obtained under such conditions, were corrected by newly developed Android-based software on the same smartphone, and protein biomarker profiles were calculated. The indirect detection of recombinant bovine somatotropin (rbST) in milk extracts based on altered biomarker profile of anti-rbST antibodies was selected as a real-life challenge. RbST-treated and untreated cows clearly showed reproducible treatment-dependent biomarker profiles in milk, in excellent agreement with results from a flow cytometer reference method. In a pilot experiment, anti-rbST antibody detection was multiplexed with the detection of another rbST-dependent biomarker, insulin-like growth factor 1 (IGF-1). Milk extract IGF-1 levels were found to be increased after rbST treatment and correlated with the results obtained from the reference method. These data clearly demonstrate the potential of the portable protein microarray concept towards simultaneous detection of multiple biomarkers. We envisage broad application of this ‘protein microarray on a smartphone’-concept for on-site testing, e.g., in food safety, environment and health monitoring.

## Introduction

On-site detection platforms have a broad significance for food safety, environment and health monitoring, as they allow the analysis of samples on the spot and the initiation of immediate measures depending on the outcome of the test. For instance, in food safety control, on-site platforms can be used to detect the presence of contaminants, such as veterinary drug residues in milk, directly at the farm prior to milk collection. In environmental monitoring, toxins, heavy metals or endocrine disrupting chemicals can be analyzed directly at the sampling site and in health care, disease biomarkers can be monitored using an on-site testing platform as a point-of-care (POC) device [1, 2]. In recent years, several on-site platforms have been developed, for instance for food allergen testing [3–5], monitoring of polycyclic aromatic hydrocarbons (PAHs) in river water [6], detection of heavy metal contamination in water [7, 8], red and white blood cell analysis [2, 9] and diagnosis of infectious diseases [2, 10–12]. Topol summarized that “the 2010s will probably be known as the era of digital medical devices” [13], but these recent developments demonstrate a broader significance of on-site detection platforms beyond disease diagnosis. Another trend within the area of on-site monitoring is the use of standard smartphones, equipped with simple attachments for analysis [2, 9, 14, 15]. These attachments usually contain simple electrical or optical components to enable detection or imaging analysis of the sample, the latter using the built-in camera of the smartphone. Using smartphones as a detection platform offers the opportunity to immediately transmit the results, including time and GPS-location data, to food safety authorities, environmental monitoring centers or medical specialist doctors via wireless network connection [10].

For any on-site testing application area, the simultaneous analysis of multiple analytes is advantageous, because it saves analysis time, money and sample volume. Especially when considering the diverse conditions in which on-site analyses take place, the inclusion of negative and positive controls in the very same test are crucial to ensure valid test results. We present here, as an example of a widely applicable multiplex testing concept, a protein microarray for biomarker detection in milk on a standard smartphone that includes positive and negative controls. Design features are 48 array spots, thus allowing replicate measurements of multiple biomarkers, and built-in positive and negative quality controls.

**Fig 1:**
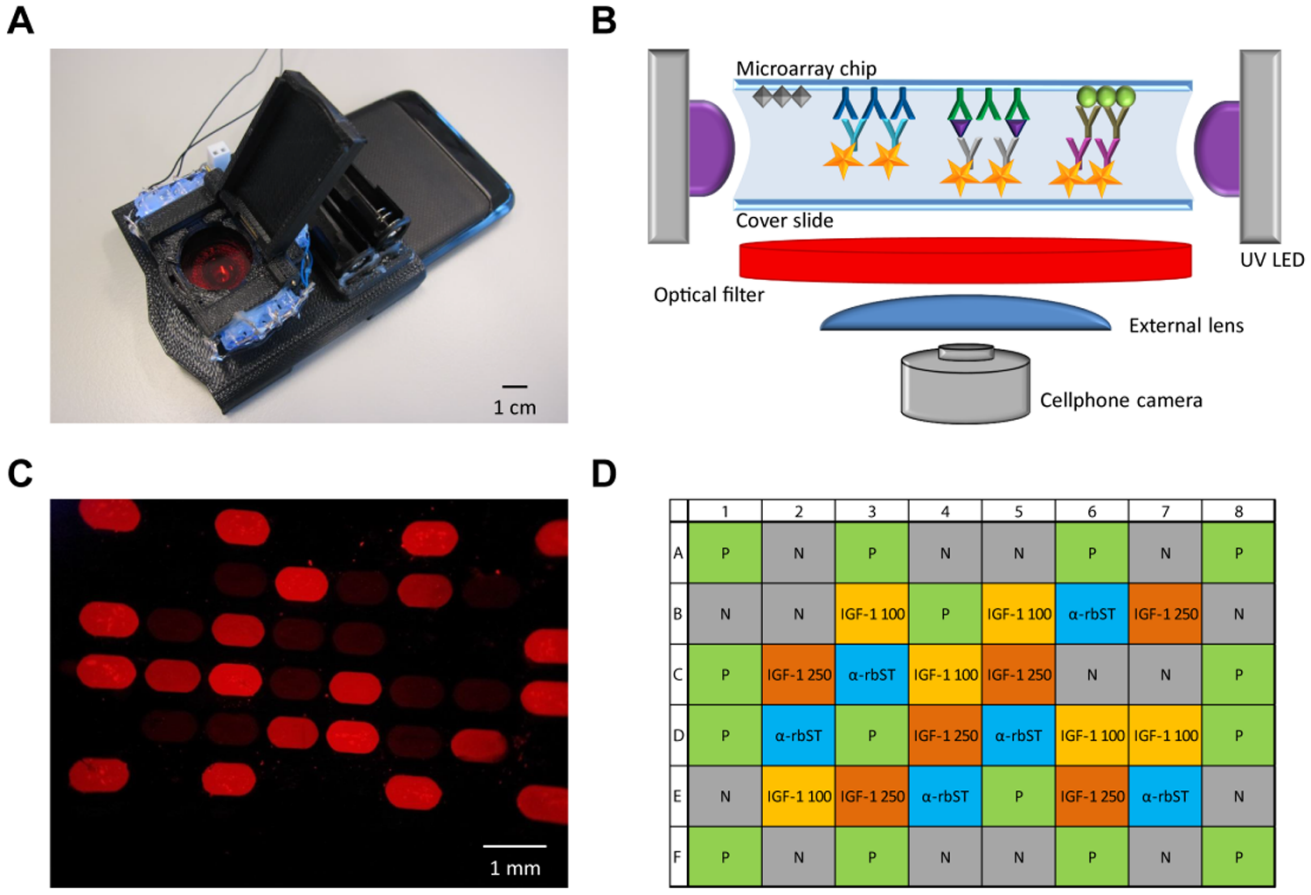
Smartphone with fluorescence microscope attachment, detection setup and microarray image. (A) Photograph of the 3D-printed microscopic imaging attachment on the smartphone that was used for analysis. (B) Setup of the smartphone biomarker detection platform. (C) Exemplary microarray image obtained using the smartphone fluorescence microscope. (D) Microarray layout for spotting of four different proteins: recombinant bovine somatotropin (rbST) for detection of the biomarker anti-rbST antibody (α-rbST, blue), anti-insulin-like growth factor-1 (IGF-1) antibody (spotted in two concentrations: 100 μg mL^-1^ and 250 μg mL^-1^) for detection of the biomarker IGF-1 (IGF-1 100, yellow; IGF-1 250, orange), ovalbumin as a negative control (N, grey) and sheep IgG as positive control (P, green).

We selected the biomarker-based detection of recombinant bovine somatotropin (rbST) abuse in milk sample extracts from dairy cows as a challenging real-life showcase. RbST is a proteohormone that can be used to increase milk production, but its administration is illegal in the EU [16] and of consumer’s concern in the USA. To detect rbST abuse, it has been shown that rbST-dependent protein biomarkers in serum and milk of dairy cows can be monitored, but levels are typically low (10 ng mL^-1^ range) [17–19]. Here, we analyze low levels of the biomarker anti-rbST antibodies in milk sample extracts by a fluorescence immunoassay microarray. The array is conveniently imaged using a smartphone equipped with a 3D-printed opto-mechanical attachment originally designed as a fluorescent microscope for particle analysis (Fig 1A) [20]. The captured images can be instantly analyzed using the custom-developed Android application ‘Spot-An-Array’ on the same phone, and the results were critically compared with laboratory-based reference methods. For many biomarker-based methods in sports doping and veterinary control, a single biomarker is not sufficient for drug abuse detection because of high inter-individual variations [18, 21, 22]. Therefore, in a preliminary experiment, anti-rbST antibody detection was multiplexed with the detection of another rbST-dependent biomarker, namely insulin-like growth factor 1 (IGF-1). Data from this pilot demonstrate the potential of the microarray towards simultaneous detection of multiple biomarkers.

## Materials and Methods

### Reagents

Monsanto rbST standard was obtained from the National Hormone & Peptide Program of Dr. Parlow (Torrance, CA, USA). Posilac 500 mg single-dose syringes and syringes with only the slow-release formula were purchased from Monsanto Company (St Louis, MO, USA) and Ely Lilly and Company (Indianapolis, IN, USA). Rabbit polyclonal anti-IGF-1 antibody (H-70; sc-9013), biotinylated donkey polyclonal anti-sheep IgG antibody (sc-2475) and biotinylated polyclonal goat anti-bovine IgG antibody (sc-2420) were bought from Santa Cruz Biotechnologies (Santa Cruz, CA, USA). Sheep IgG (013-000-003) was obtained from Jackson Immunoresearch Europe Ltd (Suffolk, UK) and albumin from chicken egg white (ovalbumin), N-hydroxysuccinimide (NHS), 2-(N-morpholino) ethane sulfonic acid (MES), 1-Ethyl-3-(3-dimethylaminopropyl)carbodiimid (EDC), ethanolamine hydrochloride and tris(hydroxymethyl)-aminomethan (Tris) were bought from Sigma-Aldrich (St. Louis, MO, USA). MultiScreen HTS filter plates (pore size 1.2 μm) and Amicon Ultra 0.5 mL 30 K centrifugal filter units were purchased from Millipore (Billerica, MA, USA). Polycarboxylate hydrogel-coated borosilicate glass chips (24.9 x 24 x 0.13–0.16 mm) were from XanTec bioanalytics GmbH (Düsseldorf, Germany). Biotinylated polyclonal goat anti-IGF-1 antibody (BAF291) was obtained from R&D Systems (Minneapolis, MN, USA) and Streptavidin-conjugated Quantum Dots (QD) were from Life Technologies (Grand Island, NY, USA). Streptavidin-conjugated R-phycoerythrin was bought from Prozyme (San Leandro, CA, USA) and color-encoded carboxylated SeroMAP microspheres (microsphere sets 050 and 067) from Luminex (Austin, TX, USA). Prot G HP SpinTrap columns and 30000 MWCO VivaSpin500 units were obtained from GE Healthcare Europe GmbH (Diegem, Belgium). Sodium chloride (NaCl), monosodium phosphate monohydrate (NaH_2_PO_4_ x H_2_O), potassium dihydrogen phosphate (KH_2_PO_4_), Tween-20 and glass microscope cover slides (rectangular: 24 x 32 mm, thickness 1) were obtained from VWR International (Amsterdam, The Netherlands) and glycine was from Duchefa (Haarlem, The Netherlands). Sodium hydroxide (NaOH), sodium hydrogen carbonate (NaHCO_3_), disodium hydrogen phosphate dihydrate (Na_2_HPO_4_ x 2 H_2_O), acetic acid and hydrochloric acid (HCl) were purchased from Merck (Darmstadt, Germany). Sodium dodecyl sulfate (SDS) was obtained from Serva (Heidelberg, Germany).

### Buffers and solutions

Phosphate-buffered saline (PBS; 154 mM NaCl, 5.39 mM Na_2_HPO_4_, 1.29 mM KH_2_PO_4_, pH 7.4), PBST (PBS, 0.05% v/v Tween-20), PBSTB (0.1% w/v BSA in PBST). Antibody purification: binding buffer (20 mM NaH_2_PO_4_, pH 7.0), elution buffer (0.1 M glycine-HCl, pH 2.7), neutralizing buffer (1 M Tris-HCl, pH 9.0); microarray spotting: elution buffer (1 M NaCl, 0.1 M NaHCO_3_, pH 10.0), activation buffer (0.1 M NHS, 0.1 M MES, 0.5% (w/v) EDC) washing buffer (5 mM acetic acid), coupling buffer (50 mM MES, pH 5.0), quenching buffer (0.5 M ethanolamine hydrochloride, pH 8.5); sample preparation: glycine solution I (GS I; 27.5 mM glycine, pH 0.5 adjusted with HCl); glycine solution II mixture (GS II; 230 mM glycine, 250 ng mL^-1^ IGF-2, 0.015% SDS (w/v), pH 10 adjusted with NaOH); glycine solution III mixture (GS III; 340 mM glycine, 250 ng mL^-1^ IGF-2, 0.015% SDS (w/v), pH 10 adjusted with NaOH).

### Instruments

The continuous flow microspotter was from Wasatch Microfluidics (Salt Lake City, UT, USA) and the Samsung Galaxy SII smartphone (dimensions: 12.5 cm x 6.5 cm x 0.9 cm) was obtained from Amazon.com, Inc. (Seattle, WA, USA). The smartphone fluorescence microscope attachment (dimensions: 8.2 cm x 8 cm x 3cm) was constructed as described in [20]. In short, the mechanical components, such as the smartphone holder, the sample tray and the lid were made from thermoplastic using a 3D printer from Stratasys (Eden Prairie, MN, USA). For fluorescence excitation, twelve 380 nm UV LEDs, obtained from Parts Express (Springboro, OH, USA), were positioned around the sample tray. A 610 nm long pass filter (25 mm diameter) and an aspherical lens (focal length 8 mm), both obtained from Thorlabs (Newton, NJ, USA), were used to filter the fluorescence light and improve the imaging field of view, respectively. The FLEXMAP 3D flow cytometer for the reference method was from Luminex (Austin, TX, USA).

### Sample material

Milk samples were collected from controlled *Bos taurus* animal treatment experiments as described before [17]. In brief, milk samples were from two independent animal experiments. For animal study 1, permission (EC2010-21) was obtained from the Ethical Commission of the Animal Science Group of Wageningen University and Research Centre in Lelystad (The Netherlands). Permission for animal study 2 (EC2007/71) was obtained from the Ethical Commission of the Faculty of Veterinary Medicine of Ghent University (Belgium) on basis of the Dutch law on animal studies (Wet op de Dierproeven). In experiment 1, ten Holstein dairy cows of different age (1–7 years) were divided into a treatment and a control group. After a 2-week adaption period, 8 cows from the treatment group received 500 mg rbST in a slow-release formula every second week for 8 weeks and the 2 remaining cows of the control group were treated with the slow-release formula only. In animal experiment 2, eight 5-year-old Holstein cows were divided into two groups of 4 animals and were treated with rbST in slow-release formula or slow-release formula only in the same manner as in the first experiment. Additionally, they received another injection every week for two weeks directly after the first 4 injections. Milk was sampled weekly and kept at -20°C until further use. For the here presented study, four different milk samples from four different animals were chosen for analysis. From each of the two animal studies, one milk sample from an untreated cow and one sample from an rbST-treated cow were selected. Milk samples from rbST-treated cows were obtained after two rbST injections (three to four weeks after beginning of the treatment period), a timepoint at which substantial anti-rbST antibody presence was demonstrated previously [17].

### Antibody purification

Rabbit anti-IGF-1 antibodies (H-70) had to be purified from their storage solution to avoid interference with stabilizing proteins during protein spotting. For purification, Prot G HP SpinTrap columns were used according to the protocol of the manufacturer followed by membrane ultrafiltration using 30 kDa MWCO centrifugal filter units. Protein concentration was determined with the NanoDrop.

### Microarray chip spotting

Before protein spotting, polycarboxylated hydrogel-coated borosilicate glass chips were activated. Therefore, they were kept in elution buffer for 5 minutes, were washed three times in Milli Q water, activated in activation buffer for 15 minutes, washed three times in washing buffer and dried in a flow of nitrogen gas. Then, they were mounted in the continuous flow microspotter and spotted with the protein solutions. Protein concentrations were 100 μg mL^-1^ rbST, ovalbumin, IGF-1 antibody and sheep IgG and 250 μg mL^-1^ for IGF-1 antibody in coupling buffer. RbST and each of the IGF-1 antibody concentrations were spotted in replicates of 6 each. The remaining spots were allocated with 15 replicates each of positive and negative control. The spotting instrument settings were as follows: location 3, air gap 5 μL, flow time 30 minutes and rinse 2 minutes. After spotting, the remaining binding sites were quenched for 30 minutes in quenching buffer and washed three times in Milli Q water. Readily spotted chips were kept in PBST at 4°C until use.

### Milk extraction

For the protein microarray procedure, milk samples had to be extracted. Note that the development of a simplified on-site sample preparation method was not the objective of the present study yet. Therefore for the microarray, 250 μL milk sample (just a few drops) was mixed with 250 μL GS I while vortexing and kept at room temperature for 60 minutes. Thereafter, 500 μL GS II was added while vortexing and samples were filtered via a 1.2 μm pore size filter. For the reference method, 70 μL milk sample was mixed with 70 μL GS I while vortexing and kept at room temperature for 60 minutes. Thereafter, 140 μL GS III was added while vortexing and samples were filtered via a 1.2 μm pore size filter.

### Microarray procedure

The spotted microarray chip was washed with PBST and 1 mL milk extract was pipetted onto the chip and incubated for 60 minutes (S1 Fig). The chip was washed again and 500 μL antibody mixture was pipetted onto the chip surface (1:10000 biotinylated donkey anti-sheep antibody, 1:10000 biotinylated goat anti-bovine antibody, and 1:125 biotinylated anti-IGF-1 antibody in PBST) and incubated for 60 minutes. After washing the microarray chip, 500 μL 20 nM QD solution was pipetted onto the chip and incubated for 30 minutes. After a final washing step, the microarray chip was covered with a cover slide to keep the fluorescence label in an aqueous environment and for easier handling. The microarray chip was then positioned into the smartphone microscope attachment and was imaged using the ultraviolet LED-based excitation in the smartphone attachment. The phone camera was set to ‘night mode’ for increased sensitivity.

### Image analysis

To be able to analyze the images instantly on the phone, we developed a custom Android application called “Spot-An-Array” using SDK tools 24.0.2 from The Android Open Source Project (source.android.com) and ImageJ plugin [23]. The aim of the image analysis is to determine the luminance systematically for every spot and calculate luminance values of the spot area in a standardized way. Since all images showed spatial aberrations (see e.g., Fig 1C), it was necessary to transform each image to a standardized image from which the luminance values could be calculated. In a first step, the positive control spots were detected automatically by finding connected illuminating areas of a certain minimum size. Since the positive control spots were always at the same location, their centroid was used to perform a first geometric transformation of the image (i.e., affine transformation) and the image was cut to the area containing only the spots and omitting the remaining surrounding area (S2B and S2C Fig). Therewith, shearing was removed and curving was reduced. In a second transformation step (i.e. quadratic polynomial transformation), the slightly curved image was transformed in order to get a uniformly distributed grid with each spot at a distinct position (S2C and S2D Fig). After these image transformation steps, a standardized grid of centroids was used to determine luminance of each spot by taking all pixels within a defined ellipse into account (S2E Fig). The outer rows and columns of spots in the array, which only contained positive and negative controls, was only used for determining the positions of all spots. The median luminance of each spot in the inner rectangle, containing the spots for biomarker analysis as well as positive and negative controls, was reported. Then, the luminance data of respective protein spots were averaged, the background signal (negative control) was subtracted and the background-corrected luminance was normalized against the positive control on the same microarray chip. The software code can be found at http://pinguin.biologie.uni-jena.de/bioinformatik/app.html.

### Reference method procedure

For the multiplex flow cytometric immunoassay (FCIA) reference method, color-coded carboxylated SeroMAP microspheres (sets 050 and 067) were covalently coupled with 0.1 mg mL^-1^ rbST standard protein and 0.25 mg mL^-1^ purified H-70 anti-IGF-1 antibody by using the sulfo-NHS—EDC coupling chemistry as described previously [17, 18]. One hundred microliter extracted milk sample was incubated with protein-coupled microspheres for 60 minutes, microspheres were washed with PBST and 100 μL antibody mixture (1:100000 biotinylated goat anti-bovine IgG antibody and 1:125 biotinylated anti-IGF-1 antibody) were added and incubated for 60 minutes. Microspheres were washed and 100 μL 1:1000-diluted Streptavidin-R-Phycoerythrin were added and incubated for 30 minutes. Microspheres were washed and 125 μL PBST was added and microspheres were analyzed in the FLEXMAP 3D flow cytometer.

## Results and Discussion

The aim of the present study was to demonstrate a novel approach of biomarker analysis on a smartphone using a protein microarray. We selected the detection of rbST-dependent biomarkers as a challenging real-life example to proof the performance of the approach.

### Development of a smartphone-based protein microarray detection platform

**Fig 2:**
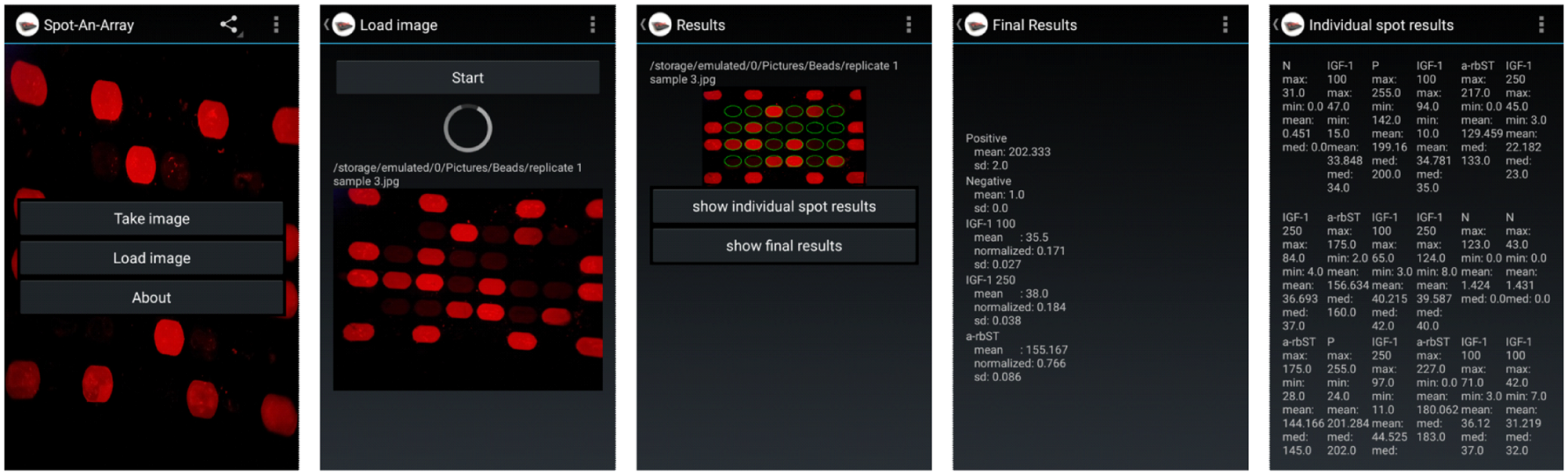
Overview of the “Spot-An-Array” Android application user interface for analysis of fluorescence images obtained using the smartphone camera. The user can decide whether a new image should be taken or an earlier taken image should be analyzed. Then, the “Spot-An-Array” Android application analyzes the selected image automatically and thereafter, a result summary or results of individual spots can be retrieved. The screenshot sequence shows the image analysis for sample number 3 (corresponding results are shown in Figs 3A and 4A).

The microarray for rbST biomarkers comprised different proteins spotted on a functionalized glass slide: rbST for anti-rbST antibody analysis, anti-IGF1 antibodies for IGF-1 detection and two quality controls, namely ovalbumin as negative control and sheep IgG as positive control. The positive control was used to monitor whether the fluorescence labelling performed properly and, moreover, provided reference points for image data analysis and normalization of all the other protein spots (S1 and S2A Figs). The microarray chip was subsequently incubated with milk sample extract, secondary antibodies and Quantum Dots, whereupon the microarray spots were fluorescently labelled depending on the amount of biomarker present in the sample extract. The microarray chip was imaged following insertion into the low-cost smartphone attachment housing having UV LEDs for fluorescence excitation, an external lens for an increased field of view, and an optical emission light filter (Fig 1A and 1B) [24]. With our custom-developed Android application “Spot-An-Array”, the user can decide whether an image should be taken or whether a previously taken image shall be analyzed (Fig 2). An exemplary microarray image as it was taken by the smartphone camera is shown in Fig 1C. The luminance of each single protein spot in the microarray images was automatically analyzed using this custom-developed Android application software code, which detected all protein microspot locations (S2C Fig) and reported median luminance values per protein spot (Fig 2). Furthermore, the software averaged the luminance data of the six respective replicate protein spots, subtracted the averaged background signal (negative control) and normalized the background-corrected luminance against the averaged positive control on the same microarray chip. The final result as well as individual spot results can be obtained from the software (Fig 2).

### Anti-rbST antibody detection on the protein microarray

**Fig 3:**
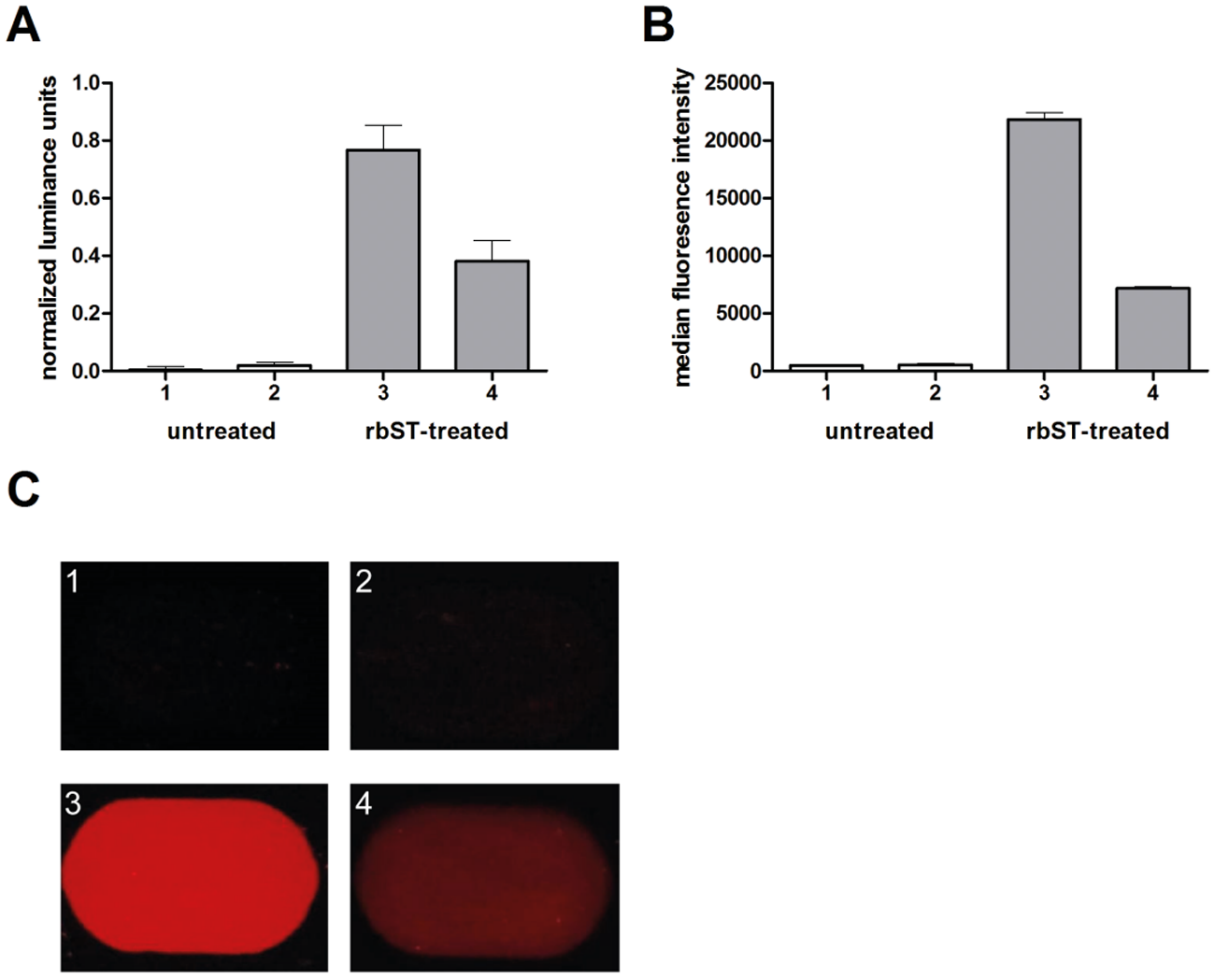
Anti-rbST antibody biomarker responses of rbST-treated and untreated cows using a protein microarray on a smartphone. (A) Normalized luminance units of the biomarker anti-rbST antibodies in milk extracts from untreated (1, 2; white bars) and rbST-treated (3, 4; grey bars) cows. Each bar represents the result obtained from a single milk sample. Error bars represent the standard deviation between the replicate spots on a single microarray chip. (B) Median fluorescence intensity obtained with the flow cytometer reference method for the biomarker anti-rbST antibodies in the same milk extracts from the same untreated (1, 2; white bars) and rbST-treated (3, 4; grey bars) cows. Error bars represent replicate measurements of the same sample. (C) Corresponding selected individual microspot images obtained with the smartphone-based fluorescent microscope for the biomarker anti-rbST antibodies.

Milk sample extracts from rbST-treated and untreated cows were tested to investigate whether these can be discriminated on the basis of their biomarker responses. Increased anti-rbST antibody levels were detected in milk sample extracts from rbST-treated cows (Fig 3A and 3C). These results are in agreement with previously found increased anti-rbST antibody presence in milk and serum after rbST treatment [17, 18, 25, 26]. Anti-rbST antibody levels obtained with the flow cytometer reference method from the same milk samples are depicted in Fig 3B and show very good agreement with the results obtained using the portable microarray approach. Note that no absolute antibody concentrations can be determined due to the lack of a protein standard of this anti-rbST antibody.

To test the reproducibility of anti-rbST antibody detection on the microarray, the same milk samples were extracted and analyzed again using another microarray chip on another day. The two replicate measurements (Table 1) show acceptable agreement. Note that the variations observed include both microarray chip-to-chip variation and day-to-day analysis variations, and can be considered as realistic performance indicators for eventual real-life implementation.

**Table 1:** Normalized luminance units of replicate measurements of anti-rbST antibodies in milk sample extracts.

sample	replicate 1	replicate 2	average
1	0,004	-0,007	-0,002
2	0,019	0,018	0,019
3	0,766	0,658	0,712
4	0,381	0,252	0,317

### Multiple biomarker detection on the microarray

**Fig 4:**
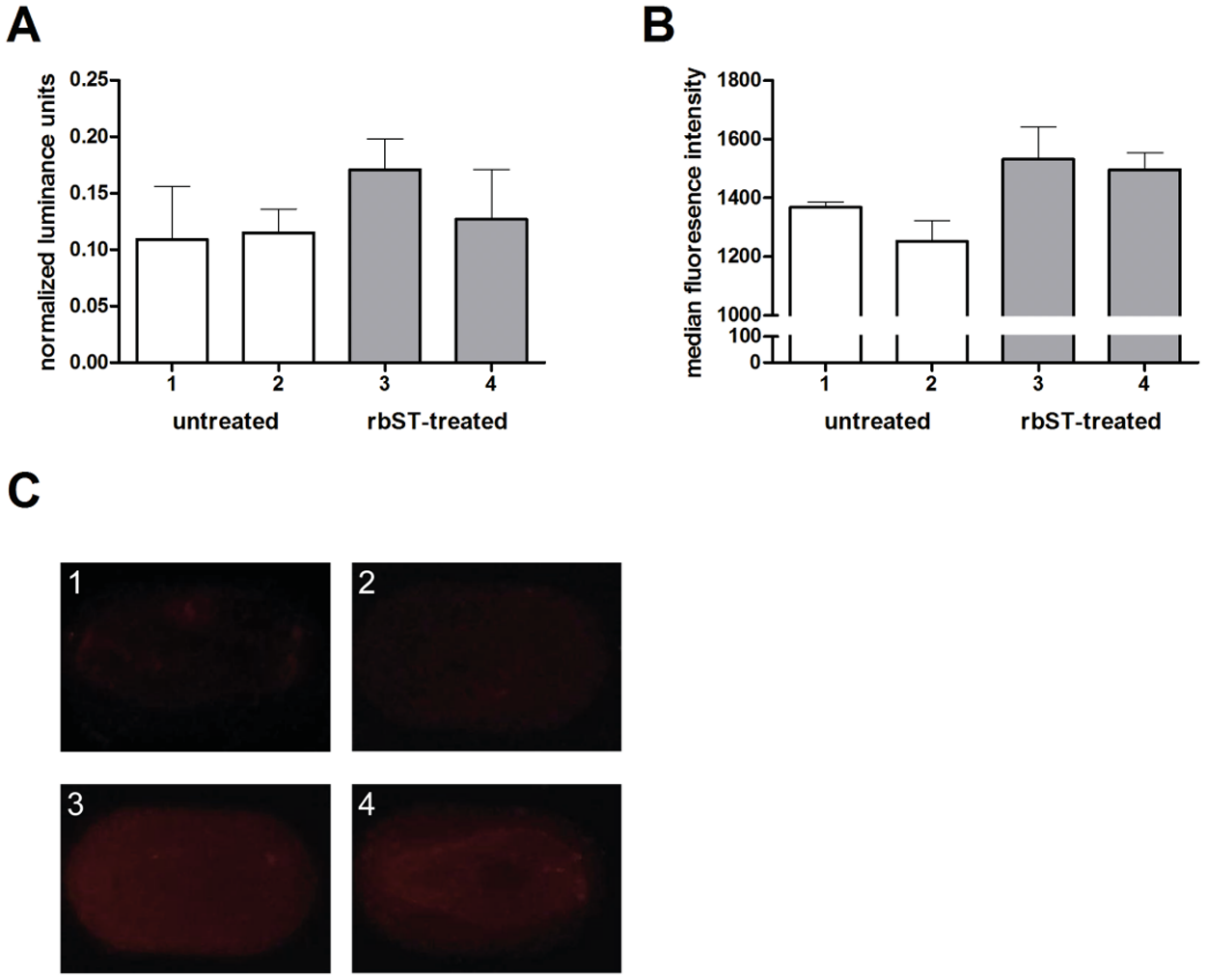
IGF-1 biomarker responses of rbST-treated and untreated cows using a protein microarray on a smartphone. (A) Normalized luminance units of the biomarker IGF-1 in milk extracts from untreated (1, 2; white bars) and rbST-treated (3, 4; grey bars) cows. Each bar represents the result obtained from a single milk sample. Error bars represent the standard deviation between the replicate spots on a single microarray chip. (B) Raw median fluorescence intensity values obtained with the flow cytometer reference method for the biomarker IGF-1 in the same milk extracts from the same untreated (1, 2; white bars) and rbST-treated (3, 4; grey bars) cows. Error bars represent replicate measurements of the same sample. (C) Corresponding selected individual microspot images obtained with the smartphone-based fluorescent microscope for the biomarker IGF-1.

To demonstrate the multiplexing capability of the microarray approach for additional rbST biomarkers in milk extracts, preliminary results were obtained for the biomarker IGF-1, which is known to be increased in milk after rbST treatment [19, 27]. Therefore, next to rbST, ovalbumin and sheep IgG, 6 locations on the microarray chip were coupled with specific anti-IGF-1 antibodies in two different concentrations respectively (Fig 1D). A sandwich immunoassay for IGF-1 detection was performed on the chip along with the measurement of anti-rbST antibodies and included positive and negative controls (S1 Fig). Preliminary results indicate that IGF-1 levels are very low in milk extracts but can be detected using the smartphone multiplex microarray approach (Fig 4A and 4C). RbST treatment increased IGF-1 levels in milk and the trends in detected signals resemble the results that were obtained using the flow cytometer reference method (Fig 4B). Nevertheless, in all tested samples, background levels were relatively high for IGF-1 due to endogenous levels, as also observed in the reference method (Fig 4B).

In the smartphone multiplex protein microarray approach, anti-rbST antibodies appeared to be more discriminative than IGF-1, similar as in more sophisticated lab-based methods [18]. Anti-rbST antibodies are a very specific biomarker and only induced after rbST treatment and not endogenously present in serum or milk otherwise [17, 18], whereas IGF-1 is always present in serum and milk at certain background levels. Daxenberger et al. demonstrated that up to 59.5% of rbST-treated cows can be detected on basis of their IGF-1 levels in milk. Anti-rbST antibodies are present in milk samples from 67% of the rbST-treated cows [17]. A combination of the results of both biomarkers can potentially increase the true-positive rate of biomarker-based rbST abuse detection, similar as being done in sports doping control [21, 22].

In order to fully validate the smartphone-based multiplex protein microarray including two (or more) different rbST-dependent biomarkers, several validation experiments will be performed in the future including reproducibility testing for IGF-1. Higher numbers of milk samples from rbST-treated and untreated cows need to be tested including the assessment of false-positive and false-negative rates, in order to validate this application for official controls. The same is necessary to account for physiological fluctuations in biomarker levels, which are expected to occur in milk as well as in serum [18, 19]. For eventual applicability of protein microarrays on smartphones in resource-limited environments, sample preparation protocols and immunoassay incubation times need to be improved. For rbST abuse detection, such an improvement would include a co-incubation step of the different assay components. The incorporation of a microfluidic device, having all assay components included [28], could minimize the required manual handling steps. Alternatively, the use of a membrane filter microarray format, in which the sample and the bioreagents are flowing through a membrane filter, may overcome diffusion-limited binding assay kinetics and thereby reduce the required incubation time to the desired minimum [29].

### Discussion

Using the camera of a smartphone as an assay readout has been reported before, for instance for the analysis of proteolytic activity of enzyme solutions in a microtiter plate format by using a hand-held UV lamp for fluorescence excitation [30]. Our approach, however, represents a generic multiplex protein analysis methodology using a simple smartphone attachment that has all required optical components integrated and therewith renders external equipment unnecessary. Compared to previous reported particle-based smartphone concepts [20, 31], the presented microarray format shows several advantages. Only a single fluorescence image is required, while previously [20], an additional white LED had to be used to locate and count the total number of particles in the dark field image. In the multiplexed microarray format, the automatically recognized positions of the positive control spots determine the location of all other protein spots, which can then be easily analyzed using automated image data analysis. Using the multiplexed microarray format, the simultaneous detection of multiple replicates of several different biomarkers is possible and even positive and negative controls can be incorporated. The fixed location of each individual spot in the array allows the discrimination of multiple biomarkers while still using the same Quantum Dot label for all markers analyzed. The continuous flow microspotter used for production of the microarray chips allows spotting 48 different protein spots at the same time (S2 Fig), therewith providing the opportunity to detect more distinct biomarkers on the same chip and/or to perform replicate analysis. In the current format, each biomarker was analyzed using six replicate spots on the microarray and the remaining spots were allocated to positive and negative controls. Furthermore, the outer rows and columns of spots comprising only positive and negative control spots were used for defining the grid for image analysis. We recommend to use a minimum of three replicate spots per analyzed biomarker, thus providing the opportunity to analyze 6 biomarkers in parallel on one chip if positive and negative controls are included. Note that the number of spots and therewith the number of potentially analyzed biomarkers may be easily increased using a microcontact printing technique.

## Conclusions

This smartphone-based detection approach for protein microarrays clearly demonstrates that it is, in principle, possible to monitor several biomarkers simultaneously at low levels in complex sample matrices such as milk extracts. Since the microarray format is broadly applicable, multiple contaminants and/or biomarkers may be analyzed in a similar manner for other food quality and safety, environmental monitoring and health care issues. The latter includes POC applications, home-diagnostics, mobile-health, or telemedicine analysis. The showcase assay presented here only required a few drops of milk and therefore this platform is very well suited for assaying small volumes, such as blood, saliva and urine. The hardware costs for such a smartphone attachment are around 140 US dollars and are therewith significantly reduced compared to the equipment costs for the reference method [31]. The housing dimensions of the attachment can be easily adapted to the rapidly changing smartphone market by simply 3D-printing a new housing; the microarray assay kit may be eventually purchased through the internet.

A smartphone-based device offers the opportunity to instantly analyze the images using the internal computing power of the phone and transmit the results to food safety authorities, environmental monitoring centers or medical specialist doctors via wireless network connection. Therewith, immediate measures can be initiated on the spot depending on the outcome of the test. We envisage that in all these application areas, cloud-based smartphone analysis networks may be established to create spatio-temporal maps of contaminant or disease occurrence and spreading, also providing an important tool for e.g., epidemiology.

## Supporting Information

S1 Fig Protein microarray immunoassay principle. Recombinant bovine somatotropin (rbST), rabbit anti-insulin-like growth factor-1 (IGF-1), sheep IgG and ovalbumin were covalently coupled to polycarboxylate hydrogel-coated borosilicate glass chips (spot size: 400 μm x 600 μm). During the milk incubation step, anti-rbST antibodies and IGF-1 bound to their respective binding partners and during the antibody mix incubation step, biotinylated goat anti-bovine, rabbit anti-IGF-1 and donkey anti-sheep bound to their respective binding partners. In the final Quantum Dot incubation step, streptavidin-coupled Quantum Dots labelled the biotinylated antibodies present on the microarray chip.
(TIF)

S2 Fig Microarray images. (A) Microarray layout for spotting of the four different proteins recombinant bovine somatotropin (rbST) for detection of the biomarker anti-rbST antibody (α-rbST, blue), anti-insulin-like growth factor-1 (IGF-1) antibody (spotted in two concentrations: 100 μg mL^-1^ and 250 μg mL^-1^) for detection of the biomarker IGF-1 (IGF-1 100, yellow; IGF-1 250, orange), ovalbumin as a negative control (N, grey) and sheep IgG as positive control (P, green). (B)-(E) Image analysis workflow showing the original smartphone camera image (B), the image after affine transformation and sizing (C) and the image after quadratic polynomial transformation (D). (E) Final image with elliptic spots, from which the median luminance is obtained. Luminance data of respective protein spots were averaged, the background signal (negative control) was subtracted, background-corrected luminance was normalized against the positive control on the same microarray chip and normalized values were plotted in Figs 3A and 4A.
(TIF)

S1 Table Raw data and normalized results corresponding to Figs 3 and 4 in the manuscript. (PDF)
