# Supplementary figures and images for: Calling Biomarkers in Milk Using a Protein Microarray on Your Smartphone
Source: PLoS One. 2015 Aug 26;10(8):e0134360. doi: 10.1371/journal.pone.0134360 (PMC4550345; doi:10.1371/journal.pone.0134360)

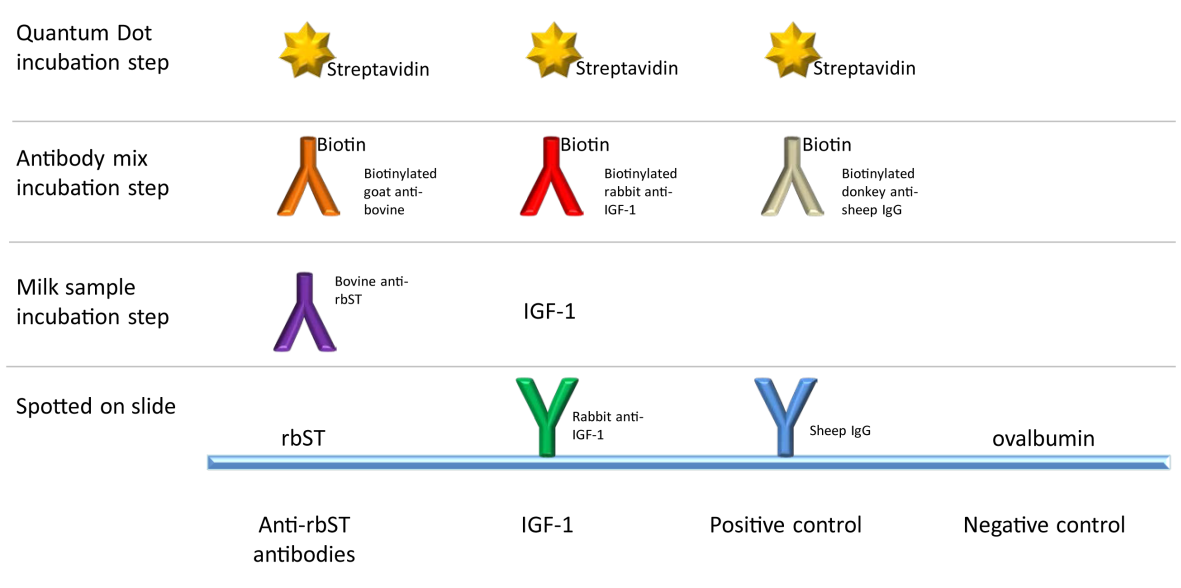

Supplement: S1 Fig — Recombinant bovine somatotropin (rbST), rabbit anti-insulin-like growth factor-1 (IGF-1), sheep IgG and ovalbumin were covalently coupled to polycarboxylate hydrogel-coated borosilicate glass chips (spot size: 400 μm x 600 μm). During the milk incubation step, anti-rbST antibodies and IGF-1 bound to their respective binding partners and during the antibody mix incubation step, biotinylated goat anti-bovine, rabbit anti-IGF-1 and donkey anti-sheep bound to their respective binding partners. In the final Quantum Dot incubation step, streptavidin-coupled Quantum Dots labelled the biotinylated antibodies present on the microarray chip. (TIF) [file pone.0134360.s001.tif]

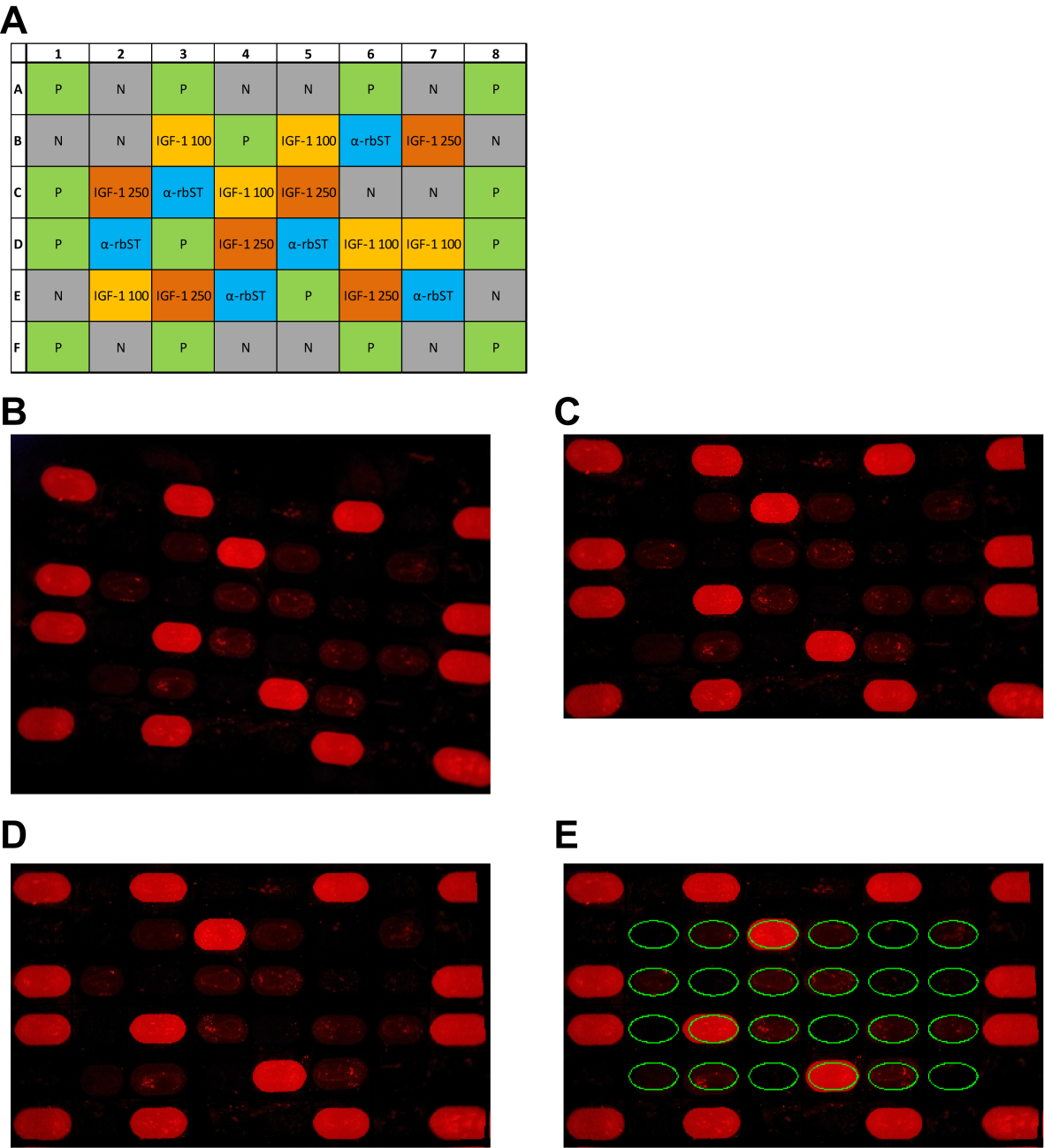

Supplement: S2 Fig — (A) Microarray layout for spotting of the four different proteins recombinant bovine somatotropin (rbST) for detection of the biomarker anti-rbST antibody (α-rbST, blue), anti-insulin-like growth factor-1 (IGF-1) antibody (spotted in two concentrations: 100 μg mL-1 and 250 μg mL-1) for detection of the biomarker IGF-1 (IGF-1 100, yellow; IGF-1 250, orange), ovalbumin as a negative control (N, grey) and sheep IgG as positive control (P, green). (B)-(E) Image analysis workflow showing the original smartphone camera image (B), the image after affine transformation and sizing (C) and the image after quadratic polynomial transformation (D). (E) Final image with elliptic spots, from which the median luminance is obtained. Luminance data of respective protein spots were averaged, the background signal (negative control) was subtracted, background-corrected luminance was normalized against the positive control on the same microarray chip and normalized values were plotted in Figs 3A and 4A. (TIF) [file pone.0134360.s002.tif]
