# Supplementary material for: Calling Biomarkers in Milk Using a Protein Microarray on Your Smartphone
Source: PLoS One. 2015 Aug 26;10(8):e0134360. doi: 10.1371/journal.pone.0134360 (PMC4550345; doi:10.1371/journal.pone.0134360)
Supplement: S1 Table — (PDF) [file pone.0134360.s003.pdf]

Intensity data corresponding to protein microarray on a smartphone results in Figures 3A

sample		positive control		negative control		anti-rbST antibodies		
		average luminance intensity	standard deviation	average luminance intensity	standard deviation	average luminance intensity	normalized luminance intensity	standard deviation
untreated	1	204.67	2.31	2.33	2.52	3.17	**0.00**	0.01
	2	201.33	2.08	1.00	1.00	4.83	**0.02**	0.01
rbST-treated	3	202.33	2.08	1.00	0.00	155.17	**0.77**	0.09
	4	185.00	8.89	0.33	0.58	70.67	**0.38**	0.07

Intensity data corresponding to protein microarray on a smartphone results in Figures 4A

sample		positive control		negative control		IGF-1 (100 μg mL^{-1})		
		average luminance intensity	standard deviation	average luminance intensity	standard deviation	average luminance intensity	normalized luminance intensity	standard deviation
untreated	1	204.67	2.31	2.33	2.52	24.33	**0.11**	0.05
	2	201.33	2.08	1.00	1.00	24.00	**0.12**	0.02
rbST-treated	3	202.33	2.08	1.00	0.00	35.50	**0.17**	0.03
	4	185.00	8.89	0.33	0.58	23.83	**0.13**	0.04

Median fluorescence intensity data corresponding to reference method results in Figures 3B

sample		anti-rbST antibodies median fluorescence intensity	standard deviation
untreated	1	**490**	70
	2	**549**	149
rbST-treated	3	**21814**	570
	4	**7177**	154

Median fluorescence intensity data corresponding to reference method results in Figures 4B

sample		IGF-1 median fluorescence intensity	standard deviation
untreated	1	**1368**	17
	2	**1252**	71
rbST-treated	3	**1532**	110
	4	**1495**	58
